# A Denoising and Fourier Transformation-Based Spectrograms in ECG Classification Using Convolutional Neural Network

**DOI:** 10.3390/s22249576

**Published:** 2022-12-07

**Authors:** Muhammad Farhan Safdar, Robert Marek Nowak, Piotr Pałka

**Affiliations:** Institute of Computer Science, Faculty of Electronics and Information Technology, Warsaw University of Technology, 00-665 Warsaw, Poland

**Keywords:** electrocardiogram, convolutional neural network, Fourier transformation, spectrograms

## Abstract

The non-invasive electrocardiogram (ECG) signals are useful in heart condition assessment and are found helpful in diagnosing cardiac diseases. However, traditional ways, i.e., a medical consultation required effort, knowledge, and time to interpret the ECG signals due to the large amount of data and complexity. Neural networks have been shown to be efficient recently in interpreting the biomedical signals including ECG and EEG. The novelty of the proposed work is using spectrograms instead of raw signals. Spectrograms could be easily reduced by eliminating frequencies with no ECG information. Moreover, spectrogram calculation is time-efficient through short-time Fourier transformation (STFT) which allowed to present reduced data with well-distinguishable form to convolutional neural network (CNN). The data reduction was performed through frequency filtration by taking a specific cutoff value. These steps makes architecture of the CNN model simple which showed high accuracy. The proposed approach reduced memory usage and computational power through not using complex CNN models. A large publicly available PTB-XL dataset was utilized, and two datasets were prepared, i.e., spectrograms and raw signals for binary classification. The highest accuracy of 99.06% was achieved by the proposed approach, which reflects spectrograms are better than the raw signals for ECG classification. Further, up- and down-sampling of the signals were also performed at various sampling rates and accuracies were attained.

## 1. Introduction

**Figure 1:**
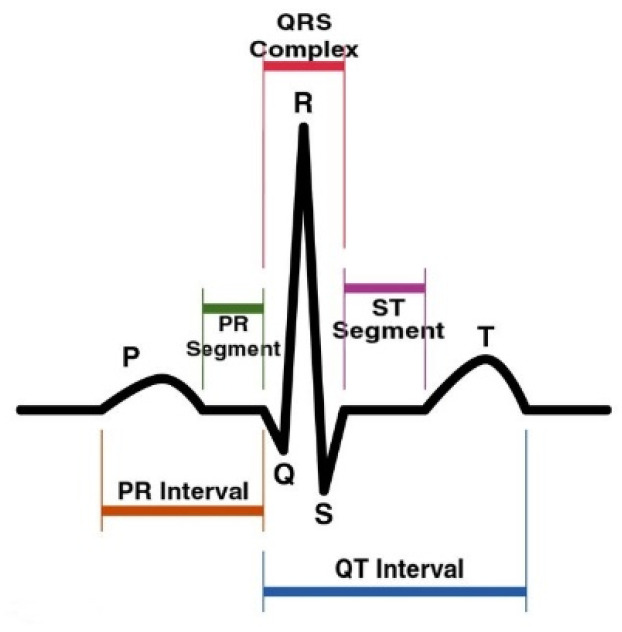
ECG cardiac cycle (image credit: Public Domain): *x*-axis (time), *y*-axis (amplitude in mV), typical duration (1 s).

Electrocardiogram (ECG) signals are non-stationary and risk-free, and using them is inexpensive to measure the electrical activity of the heart. The ECG signal consists of waves in the forms of P, Q, R, S, and T notations called one complete cardiac cycle as shown in below Figure 1, plotted as an X-Y plot where the *x*-axis represents time and voltage on the *y*-axis which shows heart activity. These are used to identify cardiac abnormalities and diseases. These waves need to be transformed for the classification model to detect heart disease i.e., a noise-free wave [1]. The ECG signals consisted of segments (as given in Figure 1) that depict the region between two waves, i.e., PR and ST. The said regions are important to know the time interval of depolarization and repolarization among heart chambers, i.e., atria and ventricles activation, and the changes in these segments are useful in heart disease prediction. Similarly, the RR interval and R-peaks are also crucial factors to determine the patient’s health status [2]. ECG is useful in finding various heart-related diseases, including cardiovascular disorder [3], heart bundle branch block [4], ST-T ischemic changes for coronary heart disease [5], atrial fibrillations due to disordered heart rhythm [6], left ventricular hypertrophy [7], and acute pericarditis [8]. Anteroseptal myocardial infarction (ASMI), considered in this study, is one of the life-threatening heart diseases that occurs due to the rupture of the volatile atherosclerotic plaque in the left anterior descending artery and can be detected through an ECG test [9].

ECG signals have some non-cardiac signals, making them noisy and thus affecting the ability to differentiate between normal and abnormal signals. The noise in the signals produces some additional frequency components, i.e., low and high frequencies. These push the vital clinical evidence beyond, and therefore, a misclassification occurs [10,11]. The noise is of diverse types, including powerline interference, muscle artifacts, baseline wanders, and electrode contact. The impact of noise on ECG is sometimes much worse, corrupting to an extent making the medical professionals impossible to study. The delays due to repeating the ECG or wrong interpretation may be harmful for the patients’ health [10,12]. A signal denoising is a crucial factor for the accurate disease classification from the ECG signals. Wavelet transformation (WT) has become famous in biomedical signals since the 1990s for giving a progressive interpretation of the data. WT extracts the features from signals by decomposing input data into smooth patterns and then performing reconstruction of the signals, which gives a better understanding [13]. WT has the following two types: continuous and discrete wavelets. The continuous WT follows the shifting and scaling mechanism of the base signal, utilizing each conceivable wavelet, therefore, it is highly redundant. The discrete WT (which is applied in this study) on the other hand pursues only the limited set of wavelets such as locations and scales, thus, more efficiently [14]. The types of noises discussed earlier, i.e., baseline wanders and powerline interference can be constricted by applying the denoising technique based on discrete wavelet as performed in [15,16]. The cleaned signal gives a better interpretation of the ECG. To apply the denoising approach, diverse wavelets from the families including Daubechies, Bior, and Coif are practiced, enhancing the signals [17]. The main reason for the preference of WT for signal denoising is that it offers a range of wavelets, i.e., Daubechies, Haar, Coif, Sym, and Bior. Therefore, there are a number of options available under a single approach to finding the best matching wavelet for the presented problem.

**Figure 2:**
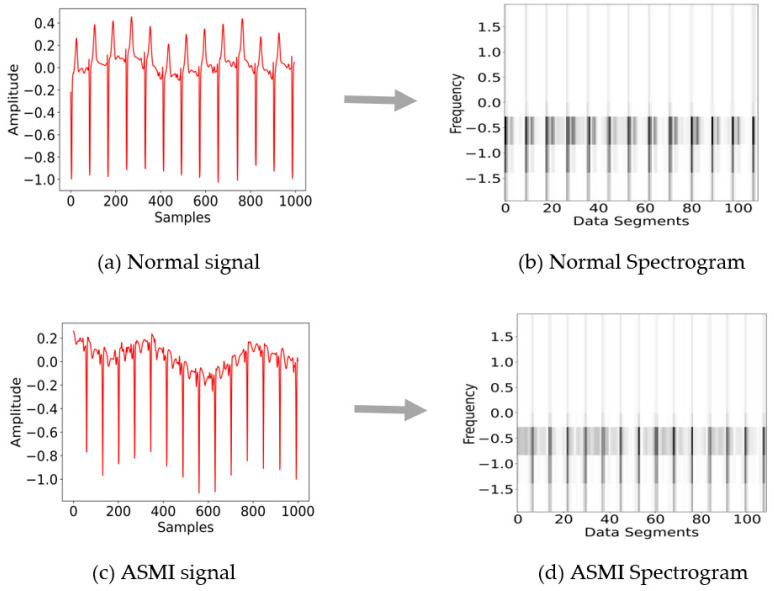
(**a**) Normal ECG signals: *x*-axis (samples/datapoints), *y*-axis (frequency in Hz); (**b**) raw signal transformed into spectrogram: *x*-axis (data segments), *y*-axis (frequency in Hz); (**c**) ASMI ECG signals: *x*-axis (samples/datapoints); (**d**) raw signal transformed into spectrograms: *x*-axis (data segments), *y*-axis (frequency in Hz).

Another approach for ECG signals analysis is Fast Fourier Transformation (FFT), which is used to transform the signals into a time-frequency domain [18]. Correspondingly, a sliding window-based version of the Fourier transformation called Short Time Fourier Transformation (STFT) deployed in the proposed study is used for the efficient signal analysis both in terms of computational time and memory. STFT is comparatively faster and has good precision, as highlighted in [19]. The other possible time-frequency transformation methods are fast Fourier transformation [20], synchro-squeezed transform [19], and continuous wavelet transformation [21]. It takes a few parameters to perform transformation, i.e., the signal data array, the length of the segments, sampling frequency as a number of samples per cycle, overlapping ratio which usually set as 25%, 50%, or 75%, and window size [22,23] as described in [24]. By applying the discussed approach, the output appears which called a spectrogram that shows time on the *x*-axis, frequency on the *y*-axis, and the color intensity represents the level of amplitude. The studies [25,26] suggest the signals are more distinguishable in the spectrograms rather than in the raw signals. In Figure 2a,c show the raw signal of the Normal (NORM) and ASMI classes, respectively, while Figure 2b,d represent the transformation of raw signals into spectrograms for both classes. A difference can be seen between the Normal and ASMI spectrograms at the signal base. There is a spread or light shadow in the ASMI spectrogram that is condensed or dark in the Normal spectrogram.

Identifying the diseases from the resultant spectrograms is not only difficult for humans but for the traditional models as well. These require narrow observation and/or computation [27]. Therefore, neural networks such as convolutional neural networks (CNN) are considered as one of the exceptional classification models. CNN takes images as an input, extracts the features, and produces the classification result with more accuracy even in a shorter time [28,29]. In recent years, it has been widely deployed for the various classification problems by the different researchers [29,30,31,32]. CNN models proposed by [33,34,35,36] used spectrograms as input to predict heart disease. In the existing ECG signal analysis, the image classification model consumes more memory and computation because of its high dimensions. Therefore, the study aimed to decrease the data size without losing vital information and affecting accuracy.

In the proposed study, the size of ECG images is reduced through denoising, unwanted frequency filtration, and generating the spectrograms by applying the STFT transformation. With this approach, comparable accuracy at a smaller number of parameters is achieved. Further, the up- and down-sampling rates of the ECG signal were performed and evaluated with the classification model on it. The novelty of the proposed work is a representation of data for the CNN model in well-presented form i.e., spectrograms. Additionally, the data size reduction aim is achieved through frequency filtration, where a simple architecture of the CNN model showed higher accuracy rather than utilizing the complex model, which ultimately requires more memory and computational power.

In Section 2, related work over various filters for signal denoising, neural networks in imaging, and spectrograms in ECG signal analysis using CNN are presented. In Section 3, the overall methodology consisting of pre-processing techniques, dataset preparation, and model architecture is described. In Section 4, experiments and their results, along with a discussion, are presented. In Section 5, a conclusion to the study is given.

## 2. Related Work

Several approaches have been adopted by the researchers to remove the noise from the signals, i.e., the authors of [37,38] applied the WT and Savitsky Golay filter, which is a signal smoothing approach. Authors in [37] additionally considered other techniques including lowpass filter, moving average filter, and gaussian and median filter for the comparative analysis. M. Gusev et al. [39] applied and compared the finite impulse response (FIR) filter, the infinite impulse response (IIR) filter, and DWT to detect the QRS peak from the signals. Study results showed that they achieved 98.2% QRS detection accuracy over the MIT-BIH Arrhythmia dataset with DWT. Likewise, generative adversarial networks (GAN) and residual networks were used by Bingxin Xu et al. [40] over ECG signals. They found GAN to be an efficient method compared to WT, the S-transform algorithm, and the stacked denoising autoencoder in terms of a better signal-to-noise (SNR) ratio. Similarly, Kan Luo et al. [41] applied the basis pursuit denoising (BPDN), low pass filter, and alternating direction method of multipliers algorithm to remove the noise from the MIT-BIH Arrhythmia dataset. Higher SNR and the lowest mean squared error (MSE) have been achieved by their implemented methods.

Neural networks are becoming essential for classification tasks and outperform other classification techniques. The authors of [30,42,43] applied CNN-based architecture models to an X-Ray imaging dataset for the classification of COVID-19 pneumonia and found the highest accuracies at 99.5%, 98.3%, and 94.5%, respectively. Cardiac vascular imaging is a non-invasive imaging test that is handy for the identification of narrowing arteries of the human heart. Ilyass Abousaleh et al. [44] performed a binary classification on the BraTS 2017 dataset using CNN architecture to classify the low- and high-grade glioma from normal images. The uppermost accuracy was achieved at 91% on the binary class problem. Likewise, Marcin Wozniak et al. [31] choose a hybrid approach of CNN and the classic artificial neural network (ANN) model, which helps CNN to select the better filters for convolutional and pooling layers and for the classification of brain tumors from computed tomography (CT) scans. The highest accuracy of 98%, has been attained by their proposed approach with 95% precision and recall. Litjens Geert et al. [32] conducted a survey of more than 80 papers presented on cardiac vascular images and neural networks. Most of the researchers utilized CT, magnetic resonance imaging, and ultrasound images as datasets. CNN-based architectures, RNN (recurrent neural network), LSTM (long short-term memory), and GAN were applied for the classification purpose. Conclusively, the neural networks are useful for disease classification, and even a few outperforming models are also under consideration by the Food and Drug Administration (FDA) for real clinical practice, as mentioned in [32]. However, further improvements can result in more achievements.

Siti Nurmaini et al. [45] evaluated an RNN-based bi-directional LSTM model for the classification of ECG in binary problems. They utilized the QT database and Lead-II of the Lobachevsky University Database for training and evaluation of the proposed model. Firstly, noise has been removed through discrete WT by applying the bior6.8 wavelet, a signal filtration approach, and then they performed the segmentation. Study results revealed 99.64% accuracy with 98.81% precision had been achieved. Yan Fang et al. [46] analyzed the ECG signals using the radial basis function (RBF) neural network on the MIT-BIH dataset. Initially, they removed the noise by practicing high pass (11 Hz cutoff) and low-pass filters. After that, they extracted the QRS segment features using the Pan Tompkins algorithm, which is a QRS complex detector [47], and then the authors applied k-means clustering for the sample screening purpose. Finally, they trained the neural network on a given dataset and achieved 98.9% classification accuracy. Dinesh Kumar Atal et al. [48] observed the ECG arrhythmia classification using the bat optimization algorithm [49] based on deep CNN (BaROA-DCNN) over the MIT-BIH dataset. Similar to the authors [45], they applied the WT for the removal of noise, and then a Gabor filter was exercised to extract the features. The BaROA-DCNN model has been trained on given dataset examples along with the other models, including the knowledge-based model, support vector machine (SVM), classical CNN, genetic algorithm-back propagation neural network (GA-BPNN), and CNN + LSTM, separately. The highest accuracy revealed by their projected BaROA-DCNN model was 93.19%, while the lowest was 85.07% for the knowledge-based model.

In this study, a more diverse approach is proposed than the above presented by applying denoising, cut-off frequencies, and STFT transformation to spectrograms with the CNN model. A similar study by Jingshan Huang et al. [34] classified ECG arrhythmias by applying the Fourier transformation named STFT and inputting the spectrograms into the CNN model. Five distinct types of heartbeat data, including normal, were extracted from the MIT-BIH arrhythmia dataset. The authors followed the comparable way as adopted in the proposed study except for the produced spectrograms image sizes, which were 256 × 256-dimensional spectrograms with a batch size of 2500. Study results revealed that 99% accuracy has been achieved by their approach. A similar but eight class problems classification has been performed by Amin Ullah et al. [35] by feeding spectrograms into the CNN model. Initially, they removed the noise from ECG signals using wavelet transformation and then applied Data Augmentation (DA), a cropping technique, to deal with the class imbalance. They produced the spectrograms by STFT transformation and resized the images to 256 × 256 dimensions for the final input to the CNN model. Study results showed 99.11% accuracy with 98.59% precision. Their technique follows the same way as trailed in this work; however, the current study’s images size, number of parameters, and batch size are lower by achieving comparable accuracy.

Guo Yang Liu et al. [36] assessed the ECG quality by applying the Stockwell-Transform (S-Transform) and generating the spectrograms as an input to the CNN model. They acquired the relevant dataset from PhysioNet CINC Challenge of 2011. Authors applied the Butterworth filter with a frequency cutoff of 0.1 to remove the noise from the signals. The online augmented scheme, along with S-transform, has been applied to the ECG signals for the purpose of better time-frequency depiction. Various approaches, including statistical features, CNN, and augmented S-transform were applied separately and within the combination. Study results showed that the highest accuracy achieved was 93.01% when the mentioned approaches i.e., AugS-Transform with CNN + Statistical Features, were applied to the given dataset. The summary of the literature review is given in Table 1 below.

## 3. Methodology

The ECG signals may contain various types of noises i.e., baseline wander and power line interference, which produces some additional frequency components i.e., low, and high frequencies and can affect the classification accuracy. Therefore, WT was considered for denoising the signals. Similarly, Fourier transformation divides the biomedical signals into time-frequency domain, through which an unwanted frequency can be filtered with the purpose of data size reduction. The key advantage of reducing data size is to present the complete data into less resolution image i.e., spectrogram which helps in memory and computational management. Spectrograms depict the data in a more distinguishable form than the raw signals i.e., recent studies [34,35,36] reveal that the data feed as spectrograms into neural networks resulted in better accuracy, which urged us to produce spectrograms using STFT.

Initially, the 12-leads ECG PTB-XL dataset was downloaded from the well-known repository physionet.org [50]. The dataset consisted of 21,837 records of 18,885 patients recorded at 100 Hz sampling rate over 10 s in length. The current study methodology into three parts. Firstly, pre-processing on the given data was applied and spectrograms were generated. Following this, datasets were prepared and finally, CNN model for classification purposes was applied. The details are given below:

### 3.1. Pre-Processing

#### 3.1.1. Denoising

The given signal data had some noise on the baseline, which affects the overall accuracy, therefore, it is necessary to clean it. The thorough experiments by the authors of [10,51], gave us the motivation to apply the WT for our purposed work to remove the noise from the signals as shown in Algorithm 1 when it retains important diagnostic data [51].
**Algorithm 1:** DenoisingStep 1: $L_{coeff}=dwt\left(data,wavelet,mode\right)$
Step 2: Vsigma=[(10.6745)×Ma. dev(Lcoeff)]
Step 3: $V_{thresh}=V_{sigma}\times \sqrt{(2\times log\;\left(len\left(data\right)\right)}$
Step 4: $F_{coeff}=\forall \;x_{i}\in L_{coeff},\;\left\{\;i=0,\;1,2\ldots n\;\right\}$
$\;thresh\left(x_{i},V_{thresh},mode\right)$
Step 5: $R_{sig}=Inverse\_dwt\;\left(F_{coeff},wavelet,mode\right)$


In Algorithm 1, initially, the list of coefficients was calculated ($L_{coeff}$) using discrete wavelet transformation (dwt) from pyWT python library [52]. The function dwt takes three parameters including signal data, wavelet as bior3.1 (as proposed work results are better at this wavelet), and the mode as periodic. The results of dwt are stored in $L_{coeff}$. Now, the sigma value ($V_{sigma}$) was considered to find the threshold by measuring the mean absolute deviation ($M_{a.\;dev}$) of the $L_{coeff}$. The constant value of 0.6745 set as given at [53], which depicts the ratio of standard deviation to ($M_{a.\;dev}$) and is a robust value of noise coefficient amplitude [54]. In the next step, the threshold value ($V_{thresh}$) was computed by multiplying the $V_{sigma}$ with the log of a given data length. On step 4, $V_{thresh}$ was applied to keep only those coefficients ($F_{coeff}$), which have greater value than the $V_{thresh}$ to remove the unwanted coefficients from the list. The thresh function was utilized from the pyWT library [52], which needed three arguments including each index of the coefficient list (found in step 1), threshold value (found in step 3), and mode as periodic. Finally, the inverse dwt was applied to retrieve the signal and stored it in resultant signal ($R_{sig}$). The inverse dwt function [52] takes the following variables as input: filtered coefficient list as $F_{coeff}$, wavelet, and the mode. The $R_{sig}$ is then stored in a NumPy file for further frequency filtration.

#### 3.1.2. Frequency Filtration

Frew frequencies were filtered to reduce data to the next level after performing the denoising approach. The study is based on the ASMI disease classification, which only focuses on the baseline signals; therefore, data that presents peak-level signals was deleted i.e., R-peaks. The various cutoff frequencies were manually tested, and value 2 Hz found as the best cutoff. At this cutoff value, vital information is retained by achieving success in data reduction aim.

**Figure 3:**
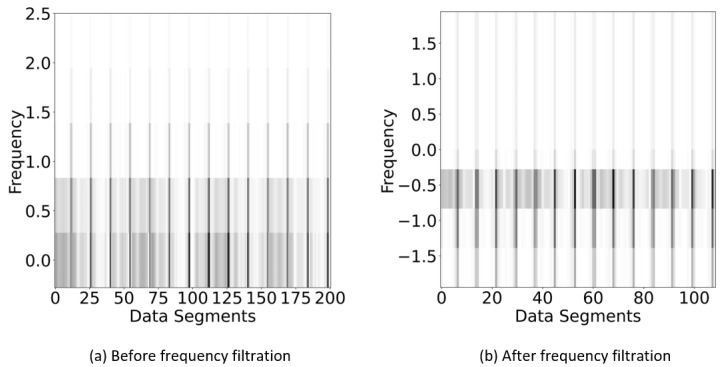
(**a**) Spectrogram before frequency filtration: *x*-axis (data segments), *y*-axis (frequency in Hz), (**b**) spectrogram after frequency filtration: *x*-axis (data segments), *y*-axis (frequency in Hz).

As shown in Algorithm 2, firstly, denoised signals data were loaded from NumPy files, which were stored during the denoising process (output of Algorithm 1). Next, FFT was computed on the given time series data and stored it into variable $x_{t}$. In step 3, sampling frequency ($S_{freq}$) of each signal (stored in variable $x_{i}$ from $x_{t}$) was calculated using the SciPy Fourier transformation frequency function. At the same time, a frequency cutoff value on the signal $x_{i}$ was applied to delete the unwanted frequencies along with the data and store the results in variable $x_{clean}$. Finally, inverse Fourier transformation was used to retrieve the $x_{clean}$ reduced signal data and stored it in variable $F_{sig}$. The data in $F_{sig}$ was further stored in a NumPy file to generate the spectrograms. In the below Figure 3a is showing spectrogram before frequency filtration, in which *x*-axis represents data segments, which means each segment contains five data points with their corresponding frequencies at *y*-axis, while Figure 3b is showing after operation with the same axis information. However, number of data segments reduced in Figure 3b because we dropped all the data points having frequencies greater than 2 Hz instead of replacing them with zero values. We considered the data segments at *x*-axis instead of time because it gives more presentable and disguisable information (among both classes) for the purpose of neural network training with improved accuracy as shown in results section. It is pertinent to mention here that, both *x* and *y* axis were completely omitted from all of figures during preparation of dataset with the aim of neural network model training because it can affect the overall accuracy.
**Algorithm 2:** Frequency filtrationStep 1: x=load_data (signal)
Step 2: $x_{t}=fourier\_transformation\;\left(x\right)$
Step 3: $\forall \;x_{i}\in x_{t},\;\left\{\;i=0,\;1,2\ldots n\;\right\}\;\;S_{freq}=fourier\_trans\_freq.\;\left(x_{i}\right)\;\;\;x_{clean}=del\;(x_{i},\;\;\;S_{freq}>2\;Hz)$
Step 4: $F_{sig}=Inverse\_fourier\_transform\;\left(x_{clean}\right)$


#### 3.1.3. Spectrograms

The spectrograms give a more distinguishable shape to the signals than the raw signals [25,26]. Therefore, spectrograms were produced after performing the denoising and frequency filtration procedures. SciPy signal library was utilized to import the STFT-based transformation for the spectrograms. The STFT takes a few arguments (as discussed in the introduction section) to generate the spectrograms. Several parameter values were tested and found few suitable by manual visualization where spectrograms give better interpretation and are more distinct. Thus, the number of FFTs (NFFT) and segments were set to 9, over looping length to half of the NFFT, and sampling frequency to 100 Hz at which signals were computed by the machine. The dimensions of the spectrograms were set to 64 × 64 with the grayscale color map and stored in the form of portable network graphics (.PNG) images.

### 3.2. Dataset Preparation

The PTB-XL signal was originally calculated at sampling rates of 100 Hz and 500 Hz. However, 100 Hz sampling rate was chosen in which each signal measured for 10 s of the length. The dataset consisted of 44 classes, out of which only a few had a higher number of samples. There was a class imbalance among the dataset classes. The whole dataset divided into two parts ASMI and normal (NORM) according to proposed work. ECG consists of 12 leads (I, II, III, aVR, aVL, aVF, v1–v6), only v1 lead data is acquired in the dataset preparation. Only those samples were inserted in the ASMI class, which originally belonged to this and the rest of the images were moved to the NORM class. Since the class imbalance occurs for the ASMI, therefore, two data augmentation approaches were applied i.e., horizontal flip and image contrast enhancement. However, it has been carefully considered to use only data augmentation approaches, which have no effect or have possible minimum on original data. Some images were also duplicated to deal with the ASMI class imbalance problem.

### 3.3. Model Architecture

**Figure 4:**
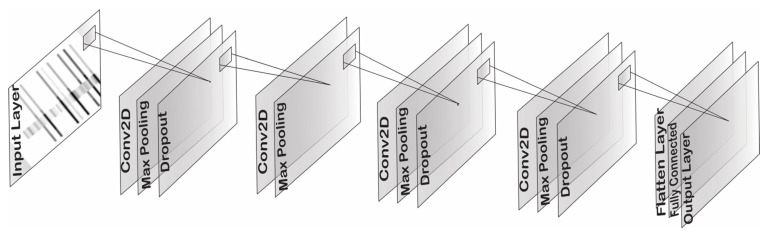
Proposed model architecture with 1 input layer, 4 convolutional layers and 1 output layer.

As discussed earlier, CNN model is one of the popular classification models for image data. Therefore, the said model is chosen for current study to perform classification over the prepared dataset. The projected architecture is given in Figure 4.

In the proposed 2-dimensional CNN model, four convolutional layers were applied. The input layer accepts a 64 × 64-dimension image as a spectrogram. The first layer has 64 filters (3 × 3) in size along with 2D Max Pooling (2 × 2) in size and 0.25 dropout to avoid overfitting. In the second layer, the filter size increased to 128 with a size of 3 × 3, and 2D Max Pooling (2 × 2) was added. The third layer was formed with an increased 256 filters (3 × 3), 2D Max Pooling (2 × 2), and 0.25 dropout. The fourth layer consisted of 64 filters (3 × 3), 2D Max Pooling (2 × 2), and 0.25 dropout. The biases and rectifier linear unit (ReLu) activation function were added in each layer. Next, the flatten layer comes up, and then a fully connected dense layer with 8 filters size appears. Lastly, one denser layer as an output layer structured with 1 filter size, sigmoid activation function, and L1 type of activity regularizer. The total number of parameters in this model are 0.52 million. In the model compilation, binary cross entropy loss with learning rate 0.001 and Adam optimizer was set. The given architecture is chosen by taking six number of layers randomly at start and attempted model training with different hyperparameters. However, the accuracy was not good as it is. Therefore, number of layers were reduced, and re-tune the hyperparameters. The best accuracy achieved at four layers and with the mentioned hyperparameters i.e., as given above.

## 4. Experimental Setting and Results

A Jupyter notebook on google research colab with a Tesla T4 GPU, 15.10 GB GPU memory, and 12.68 GB CPU RAM was set up to perform the experiments. Python as a programming language with several libraries, was used. The details of the implementation are as follows:

### 4.1. Memory Consumption

Keeping in view the data size reduction aim, firstly, two datasets, i.e., spectrograms (as discussed in the pre-processing phase) and raw signals, were created. The raw signal dataset was generated only with the denoising approach. However, the number of images (39, 908), dimensions (64 × 64), and forecolor as grayscale were the same for both datasets.

**Figure 5:**
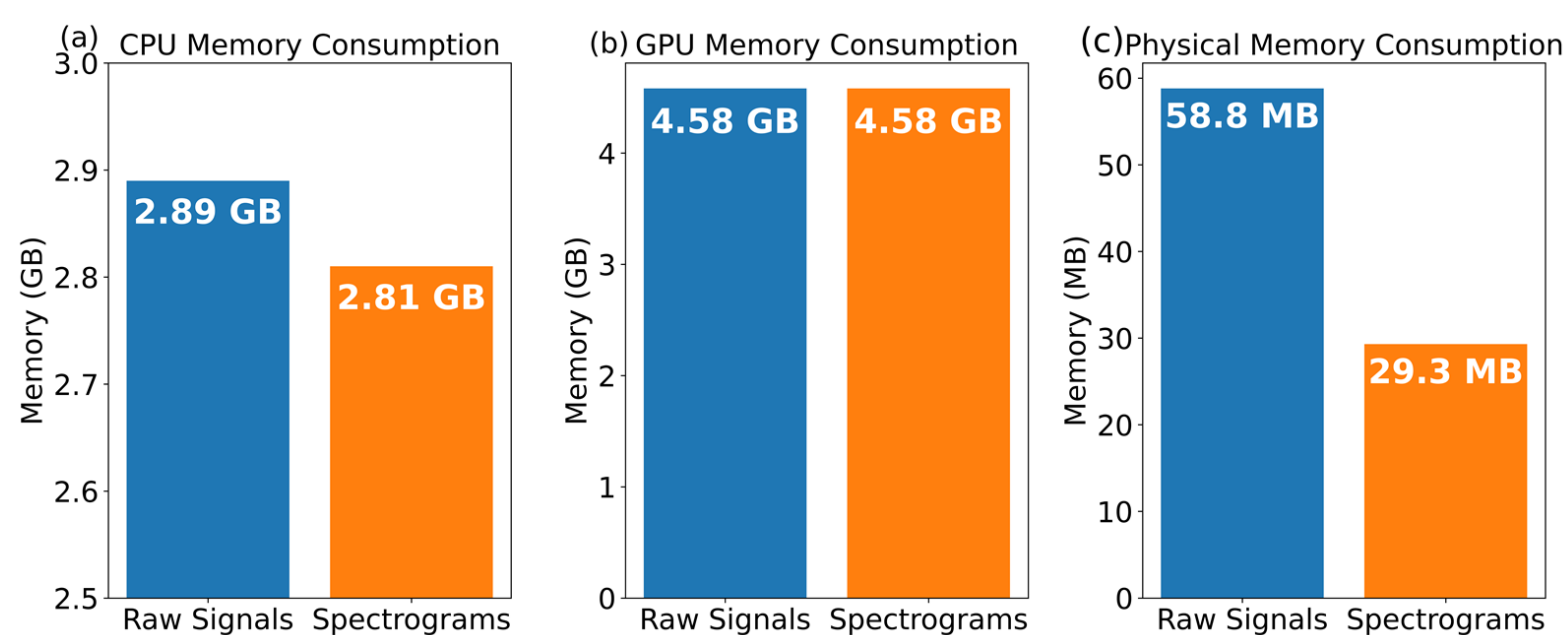
Memory proportion on both datasets: raw signals and spectrograms.

Figure 5 shows that the proposed spectrograms consume less CPU memory compared to the raw signals dataset in Figure 5a, while for the GPU memory, the memory consumption ratio is the same for both datasets in Figure 5b. However, around 50% less physical memory consumption can be seen for the spectrograms compared to the raw signals with equivalent properties in Figure 5c. The said difference is because we reduced the samples in spectrograms by applying frequency filtration. The spectrogram dataset holds a physical memory size of 29.3 MB, while the raw signals are 58.8 MB data size.

### 4.2. Model Evaluation

The CNN model was trained and evaluated on both datasets, including spectrograms and raw signals, separately. The hyperparameters were tuned as per the requirements. The batch size was set to 256 for both training and validation, along with 500 epochs and 10 steps per epoch. It is pertinent to mention here that the exact model architecture (as described earlier) and hyperparameters were utilized for both datasets.

**Figure 6:**
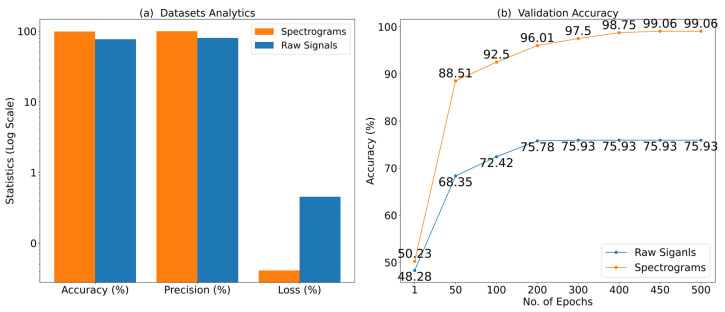
(**a**) Data analytics: accuracy, precision, and loss in log scale for both datasets: raw signals and spectrograms, (**b**) validation accuracy: *x*-axis (no. of epochs), *y*-axis (accuracy) for both datasets: raw signals and spectrograms.

Figure 6 shows the overall performance and validation accuracy for the two datasets. The accuracy, precision, and loss on the spectrograms dataset were 99.06%, 100%, and 0.04, respectively, all are in the logarithmic scale. One can see that accuracy, precision, and loss are better than the raw signals’ dataset as represented in blue bars. Similarly, in the right-hand line graph, the validation accuracy is given at different epochs for both datasets. The improved convergence curve can be seen for the spectrogram’s dataset at each next epoch, i.e., top accuracy is 99.06% at 500 epochs. The accuracy on the raw signals dataset goes into the 70s at 100 epochs but then does not improve at all.

### 4.3. Learning Rate

The CNN model achieved 99% accuracy on the spectrograms dataset; therefore, we decided to further evaluate the various learning rates. An increment and decrement in the learning rate from 0.001 were considered, the point at which the highest performance was obtained. Various learning rates such as 0.1, 0.01, 0.001, 0.0001, and 0.00001 were considered here, the rest of all other hyperparameters and model architecture kept the same as it was previously.

**Figure 7:**
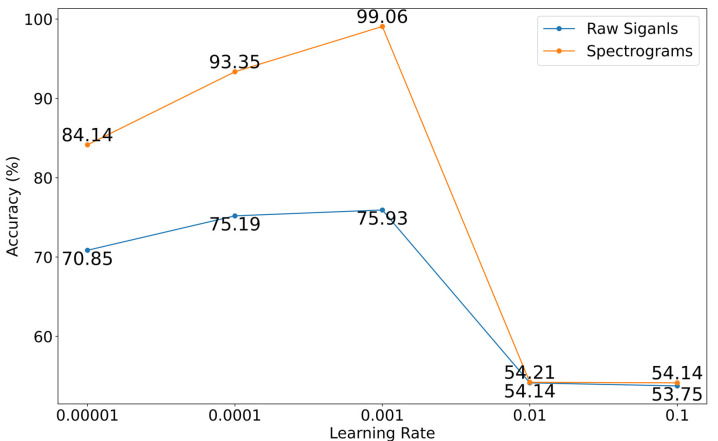
Learning rate evaluation on both datasets: raw signals and spectrograms: *x*-axis (learning rate), *y*-axis (accuracy).

Figure 7 shows the accuracy of both datasets at various learning rates. The optimal learning rate was found as 0.001 for both datasets; beyond this limit, i.e., at 0.1 and 0.01, the accuracy fell below 60%, which is the least. Similarly, when the rate goes beyond the optimal learning rate i.e., 0.001, the accuracy again showed a dropping trend.

### 4.4. Sampling Rate

**Figure 8:**
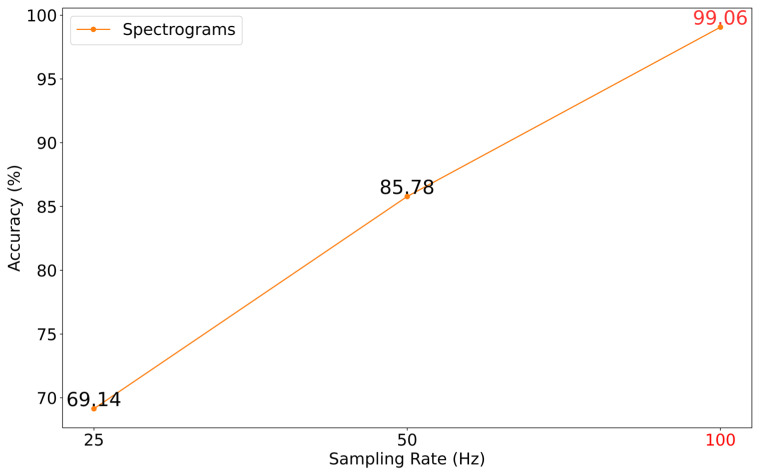
ECG sampling rate influence (down sampling) on algorithm quality: *x*-axis (sampling rate), *y*-axis (accuracy).

The well-known wfdb-python package was utilized to perform up- and down-sampling. Initially, the signals were sampled at 100 Hz, as shown in Figure 8 with red color. Signals were recalculated at a distinct sampling rate, i.e., 25, 200, 600, and 1000, to evaluate the accuracies. The same model architecture and dataset (spectrograms) were utilized for all the sampling rate experiments.

In Figure 8, less accuracy can be seen at 25 Hz and 50 Hz sampling rates. Besides the above figure, we also performed up-sampling. We checked if interpolation (up-sampling) was able to make our analysis better. For 200 Hz, we obtain 71.32% accuracy; for 400 Hz, 800 Hz, 600 Hz, and 1 kHz, we obtain 82.34%, 66.32%, 81%, and 64.21% accuracy, respectively. We concluded that adding or deleting dummy data among the original signals cannot give performance equal to the original signals.

### 4.5. Comparison with Existing Methods

The proposed approach is compared with present methodologies in terms of performance and the classification model. To prevent unfairness, only those studies were selected that had quite similar implementation approaches as the current work. Firstly, the proposed approach is compared with studies that implemented a parallel classification model over ECG signals but did not consider the spectrograms. Mishra Abhinav et al. [37] applied custom CNN to predict the arrhythmia on denoised signals using the MIT-BIH Arrhythmia Database. Their suggested approach achieved 93% accuracy, which is lower than the proposed work results. Similarly, Dinesh Kumar et al. [48] also applied an optimization-based deep CNN model to identify the ECG signals for arrhythmia. They obtained 93.19% accuracy in their proposed work, as shown in Table 2. Both authors exercised signal-denoising approaches, which are considered in this work and are obligatory to filter the noise. However, the spectrogram was found as a necessary part from this study for better classification.

Keeping in view of the above, the proposed work was compared with the authors who followed the spectrograms approach before performing the model training. Studies by [34,35,36] applied the transformation (Fourier) to generate the spectrograms as presented in the proposed work. Table 3 shows the comparison of the proposed work with the others.

Jingshan Huang et al. [34] and Amin Ullah et al. [35] achieved 99% accuracy, while Guo Yang Liu et al. [36] obtained 93.09% accuracy using spectrograms and the CNN model. The results by [36] are somehow lower because of the less optimized statistical features and CNN architecture as mentioned by them. As shown, a comparable accuracy of 99.06% was achieved in the proposed work by applying the transformation. Moreover, the unwanted frequency filtration was also applied along with denoising for further data size reduction, as discussed earlier. The high accuracy results shows that classification model such as CNN learn and perform well on the spectrograms by extracting better features than the raw signals.

## 5. Conclusions

In this study, an accurate and diverse approach is proposed for ECG signal classification. The PTB-XL ECG signals dataset used in the current study experiments was obtained from physionet.org, a well-established medical signals platform. Firstly, a denoising method using wavelet transformation (bior3.1 wavelet) was applied, and then frequency filtration was implemented to remove the unnecessary signals by taking a specific cutoff value without losing useful information. On the resulting data, the short time fourier transformation was applied to generate the spectrograms with the grayscale color scheme. Likewise, one more dataset of raw signals was also prepared by applying a similar denoising approach only for results comparison purposes. The CNN classification model on both datasets was implemented separately using the same architecture and hyperparameters. The proposed approach achieved a higher accuracy of 99.06% than the raw signals. Additionally, the CNN model was evaluated by performing up- and down-sampling of the ECG signals’ sample rate, which depicts the fluctuating accuracies. The novelty of the proposed work is using spectrograms instead of raw signals. Spectrograms could be easily reduced by eliminating frequencies with no ECG information. Further, spectrogram calculation is time-efficient through STFT. Therefore, reduced data with well-distinguishable form was presented to CNN models. These steps make the architecture of the CNN model simple which showed high accuracy. The proposed approach reduced memory usage and computational power through not using complex CNN models. In the future, the multi-class problem will be considered from a similar area, i.e., ECG signals and will emphasize the class imbalance problem.

## Figures and Tables

**Table 1:** Summary of the literature review.

Authors	Pre-Processing Approaches	Dataset	Model	Performance (Accuracy)
Mishra A. et al. [37]	DWT, Savitsky Golay filter, Butterworth filter, moving average, Gaussian Filter, Median Filter	MIT-BIH Arrhythmia Database	Custom Convolutional Neural Networks	93%
Marjan Gusev et al. [39]	FIR, IIR, DWT	MIT-BIH Arrhythmia database	-	98.20%
Bingxin Xu et al. [40]	GAN	MIT-BIH Database	Residual Network (ResNet)	40.8526 dB (SNR) 0.0102 (RMSE)
Ruixia Liu et al. [41]	BPDN, low-pass Filter, BP-ADMM	MIT-BIH ECG database	-	16.02 dB (SNR) 0.002 (MSE)
Siti Nurmaini et al. [45]	DWT, low/high-pass filters, segmentation	QT database, Lead-II The Lobachevsky University Database	Bi-directional LSTM	99.79%
Yan Fang et al. [46]	low/high-pass filters	MIT-BIH Database	RBF neural network	98.9%
Dinesh Kumar Atal et al. [48]	DWT, Gabor filter	MIT-BIH Arrhythmia Database	Bat optimization-based Deep CNN	93.19%
Jingshan Huang et al. [34]	STFT transformation	MIT-BIH Arrhythmia Database	2D-deep CNN	99%
Amin Ullah et al. [35]	DWT, data augmentation, STFT transformation	MIT-BIH Arrhythmia Database	CNN	99.11%
Guo Yang Liu et al. [36]	Butterworth filter, Stockwell transform spectrograms, online augmentation	PhysioNet CINC Challenge of 2011	CNN	93.09%

**Table 2:** Assessment with similar classification model.

Authors	Model	Accuracy
Mishra A. et al. [37]	Custom Convolutional Neural Network	93%
Dinesh Kumar Atal et al. [48]	Bat optimization based Deep CNN	93.19%
**Proposed Work**	**CNN (with spectrograms)**	**99.06%**

**Table 3:** Comparison with spectrogram approach.

Authors	Model	Accuracy	Sensitivity	Specificity	Precision
Jingshan Huang et al. [34]	2D-deep CNN	99%	-	-	-
Amin Ullah et al. [35]	Convolutional Neural Network	99.11%	97.91%	99.61%	98.5%
Guo Yang Liu et al. [36]	Convolutional Neural Network	93.09%	97.67%	77.33%	93.67%
**Proposed Work**	**Convolutional Neural Network**	**99.06%**	**99.83%**	**100%**	**99.50%**

## Data Availability

The software, developed in Python, is available freely on http://github.com/mfarhan166/ECG-Signals-and-Spectrograms under MIT license (accessed on 5 December 2022).
